# Books Review

**Published:** 1871-03

**Authors:** 


### Books Review.

### *Bound Volume of the Journal of the Gyncaeological Society of Boston.*

We have received a very beautifully bound volume of the Gynæcological Society of Boston, a journal which has now reached its thrid volume. It is devoted to a report of the proceedings of this society, and such other matters as belong to its province. It cannot be said that it is wholly confined to Gynæcology, for its chapters on Medical Ethics have operated actively upon the profession. Massachusetts has always regarded itself as one of the oldest and most influential "towns" in this country, and that its institutions are conferred by divine gift in infallible form. It has been the province of this Journal to suggest that other towns and institutions have also been discovered, and that some progress has been made within the last three hundred years, not perhaps fully understood by the founders and conductors of those old time honored and unchangable organizations.

It is a vigorous Journal, call it Gynæcological, or otherwise, as you please; and it aims to correct, as far as possible, the prevalent impression of the Pilgrim descendents, that Plymouth Rock is the foundation of the Earth; that Boston is the "Hub;" and that Harvard College is the crowning creation, of the great Mind of God. We are much oblige for volume three, in such unapproachable style. Volume one and two would add to its appearance if placed by its side, according to the old adage, "one good turn deserves another."

We must not dismiss this Journal without more seriously acknowledging its value as an original contribution to its specialty. No othor Journal in this country is devoted exclusively to the diseases of women; and it deserves support, since no field of medicine is more productive of discovery than this. Its claims upon, and interest to the profession, is nowhere excelled; and in this may be found the success which has thus far attended its publication. All physicians alive to the growing knowledge of their professions will be interested in the contents of this Journal.

---

*Physics and Physiology of Spiritualism.* By WILLIAM A. HAMMOND, M. D. D. APPLETON & Co., New York. 1871.

This is a monograph upon the subject of Spiritualism, which should be read not only by the medical profession, but by all who are able to read the English language. Such vague, ill-defined superstitions, and irrational views upon the subject of spiritualism, prevail in the community, that it would seem as though the Salem Witchcraft tragedies were soon to be again repeated. That the phenomena observed in so-called spiritualism should be regarded by thinking and otherwise sensible persons as due to supernatural causes, cannot be explained, other than as arising from a natural tendency in the minds of men to ascribe to supernatural agencies those events, the causes of which are beyond their knowledge. These phenomena have been observed at various times from the earliest history of mankind. Possessed of devils, witches, mesmerism, spiritualism, &c., &c., are some of the names it has received. It has been exhibited by various denominations of men, priests, witches, magicians, mesmerisers, somnambulists, ecstatics, hysterical persons and mediums, even down to the present age, "the skepticism of men not having reached that condition which admits of no belief without adequate proof."

Our author says: "There have always been, and probably always will be, individuals whose love for the marvelous is so great, and whose logical powers are so small, as to render them susceptible of entertaining any belief, no matter how preposterous it may be; and others, more numerous, who, staggered by facts which they cannot understand, accept any hypothesis which may be offered as an explanation, rather than confess their ignorance." Spiritualism, mesmerism, witchery, and the kindred "sciences" (?) are most satisfactorily treated in this little volume; and we think it should be forced upon the public, by a sort of missionary effort in the same way, if necessary, that religious tracts are distributed. Some rational views as to the causes of the miraculous phenomena which have been observed during the history of the world, would be of as much value to civilization as any truth in science, religion, law or medicine, and this book will aid in obtaining them.

